# Mitochondrial Fostering: The Mitochondrial Genome May Play a Role in Plant Orphan Gene Evolution

**DOI:** 10.3389/fpls.2020.600117

**Published:** 2020-12-03

**Authors:** Seth O’Conner, Ling Li

**Affiliations:** Department of Biological Sciences, Mississippi State University, Mississippi State, MS, United States

**Keywords:** mitochondrial genome, orphan gene, plant, numt, evolution

## Abstract

Plant mitochondrial genomes exhibit unique evolutionary patterns. They have a high rearrangement but low mutation rate, and a large size. Based on massive mitochondrial DNA transfers to the nucleus as well as the mitochondrial unique evolutionary traits, we propose a “Mitochondrial Fostering” theory where the organelle genome plays an integral role in the arrival and development of orphan genes (genes with no homologs in other lineages). Two approaches were used to test this theory: (1) bioinformatic analysis of nuclear mitochondrial DNA (Numts: mitochondrial originating DNA that migrated to the nucleus) at the genome level, and (2) bioinformatic analysis of particular orphan sequences present in both the mitochondrial genome and the nuclear genome of *Arabidopsis thaliana*. One study example is given about one orphan sequence that codes for two unique orphan genes: one in the mitochondrial genome and another one in the nuclear genome. DNA alignments show regions of this *A. thaliana* orphan sequence exist scattered throughout other land plant mitochondrial genomes. This is consistent with the high recombination rates of mitochondrial genomes in land plants. This may also enable the creation of novel coding sequences within the orphan loci, which can then be transferred to the nuclear genome and become exposed to new evolutionary pressures. Our study also reveals a high correlation between the amount of mitochondrial DNA transferred to the nuclear genome and the number of orphan genes in land plants. All the data suggests the mitochondrial genome may play a role in nuclear orphan gene evolution in land plants.

## Introduction

Our depth of understanding organism genomes has progressed with the advancement of sequencing technology. Throughout all domains of life, there is thought to be some level of species-specific genes called orphan genes (Tautz and Domazet-Lošo, 2011). Around 5–15% of an organism’s genes are estimated to be orphans (Arendsee et al., 2014). The function of orphan genes can range from negligible to necessary; mutants of some orphan genes in Drosophila cause lethality (Reinhardt et al., 2013). Orphan genes may also explain some species-specific functionality—Prod1, a salamander specific gene, aids in limb regeneration of salamanders, the only tetrapod with this ability (Kumar et al., 2015). In *A. thaliana*, Qua Quine Starch (QQS) – the species-specific orphan gene and its network have been shown to be involved in regulation of carbon and nitrogen allocation (Li et al., 2009, 2015; Li and Wurtele, 2015; Jones et al., 2016; O’Conner et al., 2018), and several orphans have been implicated in stress response (Graham et al., 2004; Luhua et al., 2013; Qi et al., 2019). Since the discovery of orphan genes in the yeast genome in 1996 (Dujon, 1996), researchers have made strides to explain the origin of these novel genes. However, the orphan gene origin story is still under debate today (Rödelsperger, 2018).

It was previously thought the main mechanism involved in developing novel genes was duplication and subsequent sequence diversification (Tautz and Domazet-Lošo, 2011). However, as put forth by Tautz and Domazet-Lošo (2011), there seems to be more and more evidence for *de novo* evolution of genes out of previously non-coding sequences. Although there are several theories for how *de novo* genes evolve (Bennetzen, 2005), it is not clear how a previously non-coding sequence can develop into a gene, let alone a functional gene. Many speculate nuclear non-coding DNA to be the substrate through which gene evolution occurs (Bennetzen, 2005). However, it is important to consider all “nucleotide banks” (i.e., organelle genomes) when trying to pinpoint the source of new genes. Noutsos et al. (2007) determined a possible role for Numt (mitochondrial originating DNA that has migrated to the nucleus) DNA in exon evolution. This work showed a small sampling of Numt DNA had been functionally integrated into exons of nuclear genes. They proposed there were likely more Numt insertions into exon regions that were now unrecognizable due to sequence divergence. While some work has been done to show that Numts may play a role in remodeling nuclear genes (Noutsos et al., 2007), there is currently little research into mitochondrial genomes and their impact on *de novo* orphan gene evolution.

The classical evolutionary theory of endosymbiosis states that modern eukaryotic cells are the product of a symbiotic event, wherein a proteobacterium was engulfed by a larger cell which eventually became the eukaryotic cell (Timmis et al., 2004). These proteobacteria became the mitochondria, which contains its own genome. Since the endosymbiotic event, the mitochondrial genome has begun to transfer its genes into the nuclear genome (Berg and Kurland, 2000). To put into perspective just how massive this gene transfer event has been, it is thought that there were once 1,600 proteins coded within the proto-mitochondrial cell, but now in modern mitochondria, there are typically 67 proteins or less coded from the organelle genome (Berg and Kurland, 2000). This process of mitochondrial genetic material transfer has been ongoing in the form of Numts (Timmis et al., 2004). This process is also prevalent in mammals (Woischnik and Moraes, 2002; Hazkani-Covo et al., 2003; Triant and DeWoody, 2007; Jensen-Seaman et al., 2009; Calabrese et al., 2017).

Currently, mitochondrial genomes in plants are known for odd evolutionary patterns. They are large and rearranged at a high rate while having a low mutation rate (Christensen, 2013). They also contain long intergenic regions—up to 98% (Kubo and Mikami, 2007; Sloan et al., 2012; Skippington et al., 2015). It has been recently shown that large changes between mitochondrial genomes in various Arabidopsis ecotypes are due to high recombinatorial activity controlled by *MSH1*, a nuclear gene responsible for suppressing recombination of mitochondrial DNA repeats (Arrieta-Montiel et al., 2009). The novelty of mitochondrial sequences in plant species is possibly due to large repeats and rearrangements, sequence influx from the nuclear and chloroplast genomes, and horizontal sequence transfer from other plant mitochondria (Sanchez-Puerta et al., 2008; Rice et al., 2013; Xi et al., 2013; Wynn and Christensen, 2019). Based on large mitochondrial nucleotide transfers to the nucleus of current species (Hazkani-Covo et al., 2010; Zhao et al., 2018; Zhang et al., 2020) as well as the mitochondrial unique evolutionary traits, we analyzed Numt and orphan gene data for six plant species and six animal species as well as a particular young gene present in both the mitochondrial genome and nuclear genome of *A. thaliana*. We propose a “Mitochondrial Fostering” theory where the plant mitochondrial genome may play an integral role in the arrival and development of *de novo* orphan genes. This process appears to be a continuation of the gene transfer events that began to occur after endosymbiosis (Berg and Kurland, 2000). Two approaches were used to test this theory: (1) Bioinformatic analysis of Numts at the genome level and (2) bioinformatic analysis of particular mitochondrial orphan sequences that are present in both the mitochondrial genome and the nuclear genome of *A. thaliana*—and one that codes for two different genes: AtMg01180 in the mitochondrial genome and At2g07667 in the nuclear genome on chromosome two.

## Materials and Methods

### Orphan Gene Analysis in *A. thaliana*, *G. max*, and *Z. mays*

Orphan gene number for the whole genome was gathered from greenphyl.org (version 4) for *G. max*, from Yao et al. (2017) for *Z. mays*, and from Arendsee et al. (2014) for *A. thaliana*. Orphans were determined for the mitochondrial genome and the chloroplast genome using OrfanFinder (Ekstrom and Yin, 2016).

### Targeting Peptide and Numt Analysis in *A. thaliana*

All analysis was performed with the *A. thaliana* genome from NCBI. Accession numbers: chromosome 1, NC_003070.9; chromosome 2, NC_003071.7; chromosome 3, NC_003074.8; chromosome 4, NC_003075.7; chromosome 5, NC_003076.8; and mitochondrial genome, NC_037304.1.

Numt analysis was performed as described in Ko and Kim (2016). In brief: using NCBI’s BLASTn, a list of Numts was generated by aligning the Arabidopsis mitochondrial genome against the Arabidopsis nuclear genome using parameters from Ko and Kim (2016). Numt fasta files were obtained using the getfasta command-line tool of the Bedtools suit, and Numts within 10,000 bps were merged together via mergefasta from Bedtools (Quinlan and Hall, 2010). A similar procedure was used for *G. max* Numt determination.

Of the Numt sequences, we randomly sampled a 17,400 nucleotides long sequence out of a large Numt. This large sequence was then cut into 100 sequences of 174 nucleotides in length using the splitfasta tool^1^. These sequences were translated into 58-aa-long “proteins”. We then randomly sampled 100 non-coding nuclear DNA sequences of 174-nt long. To do this, intergenic regions from chromosome two were collected (regions of the genome which do not code for any genes) from Araport (araport.org) and a 17,400-nt-long sequence was sampled and subsequently split into 100 sequences of 174 nucleotides in length and then each sequence was translated. Both lists were run through the TargetP server (v 1.1)^2^ to determine their putative targeting peptides. For each sequence, we received a letter that represents the presence of the potential targeting sequence (M = mitochondria, C = chloroplast, S = secreted, O = other). Data was organized in Excel. Pearson’s Chi-squared test was performed with R. For *G. max*, we compared Numt DNA with random TE sequences. TE sequence data was obtained from SoyBase^3^. Randomly generated sequences were produced from http://bioinformatics.org/sms2/random_dna.html. A flow chart of the procedure is presented in Supplementary Figure 1.

### Targeting Peptide Analysis on Orphan Genes From 33 Plant Species

Orphan gene protein FASTA sequences for 33 plant species were downloaded from greenphyl.org (version 4) and TargetP was used to predict targeting peptides. Parsing of TargetP data was performed with in-house python code that counted the number of Mitochondria (M), Chloroplast (C), and Secreted (S). Whole genome size for plant species was found at NCBI. Percentage calculation, correlation coefficient and *R*^2^ value were determined in excel. The data is summarized in Supplementary Table 1.

### Correlation of Numts and Orphan Genes in Six Plant Species

Orphan gene data for *O. sativa*, and *S. bicolor* was obtained from Yao et al. (2017), from http://www.greenphyl.org/cgi-bin/index.cgi (version 4) for *V. vinifera*. Numt length and mitochondrial genome size were obtained from Ko and Kim (2016). Correlation Coefficient and *R*^2^ value were determined in excel.

### Numt and Orphan Gene Analysis in Cetaceans

In order to determine orphan gene content—percentage of total genes that are orphans—in the six Cetacean species, we used a two-step BLAST filtering method. Proteome sequences were gathered from Uniprot^4^ for all species except the minke whale (NCBI) and the Bowhead whale^5^. First, we used local diamond BLASTp (Buchfink et al., 2015), a BLAST algorithm that can perform up to 20,000 times as fast as NCBI BLASTp, to align our query proteome to the other five Cetacean proteomes [*E*-value (Expect value) = 0.001]. The unaligned query sequence IDs were reported back to us. This gave us our initial set of prospective orphan genes. Next, we ran this list of orphans through NCBI tBLASTn against the refseq_rna database (*E*-value = 0.01). We excluded searching to mRNA from our query species. After this step, our unaligned query IDs became our final orphan gene list. Diamond BLASTp was carried out locally under default search parameters (*E*-value cutoff of 0.001) and databases were created manually. Query files for tBLASTn were run on the NCBI server with an *E*-value cutoff of 0.01.

### Mitochondrial Orphan Gene Analysis

Full genomic DNA sequences for the mitochondrial orphan genes were downloaded from TAIR. Alignment analysis was carried out on the NCBI BLASTn webserver. If two different segments of a query sequence were found in two different regions of a mitochondrial genome of another species (other than *A. thaliana*), the query sequence was considered knitted.

### Analysis of KNIT

Full-length genomic DNA sequence of KNIT (Knitted In Mitochondria: At2g07667) was downloaded from TAIR (arabidopsis.org). Alignment analysis was carried out on the NCBI BLAST webserver.

## Results

### *Arabidopsis thaliana*, *Glycine max*, and *Zea mays* Contain a Large Proportion of Orphan Genes in Their Mitochondrial Genomes

**FIGURE 1:**
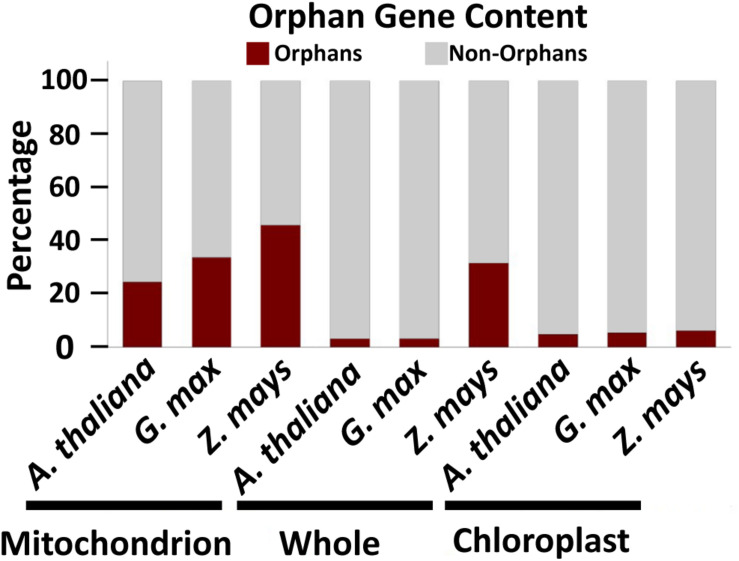
Orphan gene content in three well characterized land plants. Three plant genomes show higher orphan gene content in mitochondrial genome compared to whole genome and chloroplast genome.

To determine which cellular genomes have the highest orphan gene potential, we analyzed the number of orphan genes in mitochondrial and whole genomes (i.e., all nuclear, mitochondrial and chloroplast genomes) in three plant species with well annotated mitochondrial genomes. Some mitochondrial genomes are annotated with only homologous methods, which would exclude any orphan genes. Thus, these mitochondrial genomes were not used and we only used three plant species for this analysis. Among these three species, *G. max* has the largest number of total genes in the genome, while *Z. mays* has the largest number of mitochondrial genes, mitochondrial orphan genes, and total orphan genes (from all genomes) (Table 1). *Z. mays* also has the highest proportion of orphan genes for the whole genome and the largest proportion of orphan genes in the mitochondrial genome (Figure 1). All three species have a much higher proportion of orphan genes in the mitochondrial genome compared to the proportion of orphan genes in the whole genome (Figure 1). To understand if the phenomenon is prevalent in all organelle genomes, orphans were determined for the chloroplast genomes as well. Similar proportions of orphan genes are present in both the whole genome and the chloroplast genome for *A. thaliana* and *G. max* while *Z. mays* has a higher proportion of orphan genes in the whole genome compared to the chloroplast genome (Figure 1). As there is not a large proportion of orphan genes in the chloroplast genomes, the mitochondrial genome was focused on for the rest of this study.

**TABLE 1:** Comparison of the three cellular genomes in *A. thaliana*, *G. max*, and *Z. mays*.

	Whole genome orphan	Mitochondrion orphans	Chloroplast orphans	Total genes	Total mitochondrial genes	Total chloroplast genes
*A. thaliana*	1,169	30	4	35,387	122	80
*G. max*	2,410	24	5	73,320	71	79
*Z. mays*	20,021	63	5	63,540	137	91

*All three species have more mitochondrial orphan genes compared to chloroplast orphans.*

### Orphan Genes Are Predicted to Preferentially Locate to Mitochondria in *A. thaliana*

**FIGURE 2:**
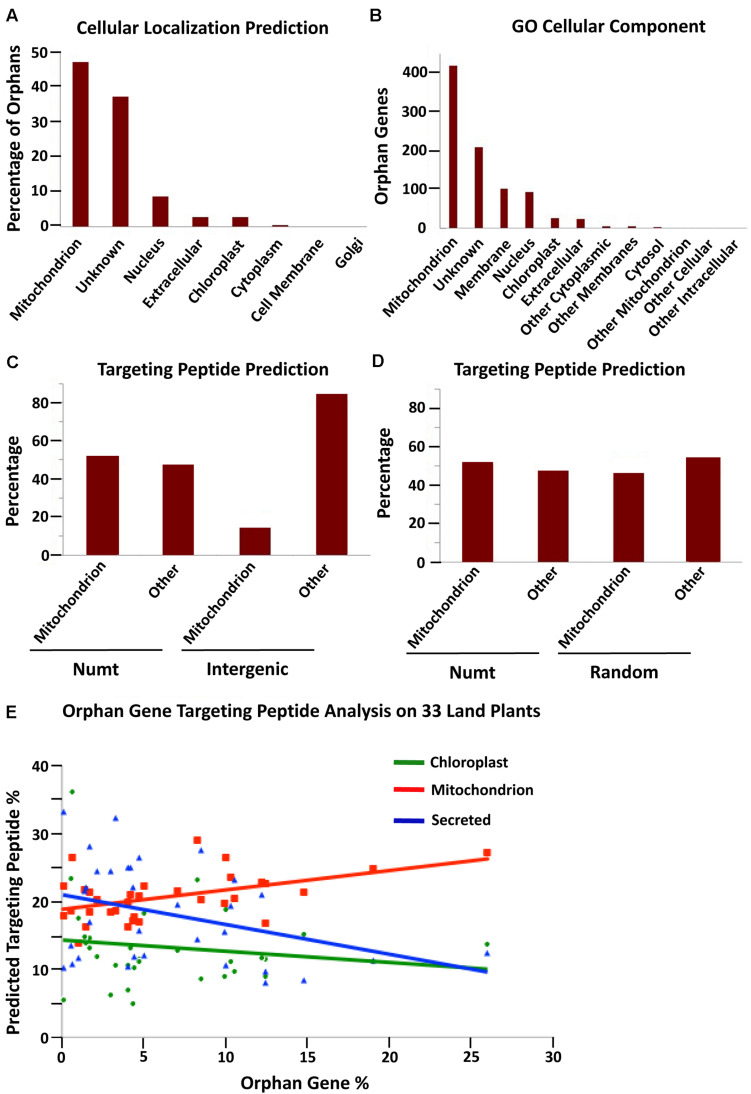
Predicted subcellular localization for *A. thaliana* orphan genes. **(A)** Subcellular localization prediction from TAIR for all *A. thaliana* 1,169 orphan genes. **(B)** Cellular component GO term analysis of *A. thaliana* orphan genes (including 852 orphan genes that have predictions). **(C)** Target peptide analysis of 100 protein sequences generated from *A. thaliana* intergenic and Numt sequences. Numt DNA is more likely to produce a mitochondrial targeting peptide than intergenic DNA (Chi-squared analysis, X-squared = 29.088, df = 1, *P* = 6.918e-08). **(D)** Target peptide analysis on *A. thaliana* Numt DNA and completely randomized DNA shows similar proclivities for mitochondrial targeting peptides (Chi-squared analysis, X-squared = 0.5002, df = 1, *P* = 0.4794). **(E)** The percentage of orphan genes with a putative mitochondrial targeting peptide shows a positive correlation with the percentage of orphan genes in the genome. The correlation coefficient for mitochondria, chloroplast and secreted targeting peptides is 0.488, –0.184, and –0.358 with *P* of 0.004, 0.305, and 0.041, respectively, *n* = 33.

As nuclear mitochondrial genes must gain a mitochondrial targeting peptide to localize back to the mitochondria (Berg and Kurland, 2000), we determined the proportion of orphan genes with predicted mitochondrial targeting peptides to determine if orphan genes preferentially code for mitochondrial targeting peptides, possibly signifying a mitochondrial origin. Subcellular localization data for *A. thaliana* was obtained from TAIR and cross-referenced with our *A. thaliana* orphan gene list (Arendsee et al., 2014). The largest portion (47%) of *A. thaliana* orphans had predicted localization to mitochondria, which was even larger than unknown localization (37.5%) (Figure 2A). To verify this analysis, the cellular component GO terms were downloaded for 852 orphan genes with the component GO terms. Again, mitochondrion had the largest number of hits (419) with unknown in second (209 hits)—percentages of 47 and 23.4, respectively (Figure 2B).

We next wanted to understand if mitochondrial-originating DNA would preferentially code for a mitochondrial targeting peptide. To do this analysis, a random selection of 100 Numt DNA segments of 174 nucleotides was translated into amino acid sequence. For comparison, a random selection of 100 nuclear intergenic sequences of 174 nt each was translated into amino acid sequence. Each data set was then run through TargetP (Emanuelsson et al., 2000) to obtain predicted target peptide sequences. The results revealed that Numt DNA (mitochondrial origin) is more likely to code for a mitochondrial targeting peptide than intergenic DNA (nuclear origin) (Figure 2C). The same technique was also used for *G. max* and similar results were obtained: Numt DNA from *G. max* was more likely to code for a mitochondrial targeting peptide compared to transposable element (TE) DNA from *G. max* (Supplementary Figure 2). The TE DNA was used for comparison as they have long been associated with orphan gene evolution (Bennetzen, 2005). Thus, orphans preferentially target mitochondria, Numt sequences preferentially code for mitochondrial targeting peptides, it is possible that some orphan genes may originate from mitochondrial sequences.

This same analysis was performed on completely randomized DNA sequences (100 sequences of 174 nt). These randomized DNA sequences showed a similar proportion of mitochondrial targeting peptides compared to Numt DNA (Figure 2D). Thus, mitochondrial-originating DNA seems to have a more random quality than nuclear intergenic DNA as both random DNA sequences and Numt DNA sequences code for a high proportion of mitochondrial targeting peptides.

To further understand how subcellular targeting may relate to orphan gene content (percentage of total genes that are orphans), all orphan gene sequences for 33 plant species (from^6^, version 4) were run through TargetP to determine their putative targeting peptides, and the percentage of mitochondrion, chloroplast, and secreted targeting peptides were determined (Supplementary Table 1). The percentage of orphans with predicted mitochondrial targeting peptides had a positive correlation with the whole genome orphan gene content of each species (Figure 2E). The other two types of predicted targeting peptides (secreted and chloroplast) had a negative correlation. Therefore, plants with more orphan genes have a higher proportion of orphans with predicted mitochondrial targeting peptides. Since mitochondrial originating DNA can create ORFs that preferentially code for mitochondrial peptides (Figure 2C and Supplementary Figure 2), an increase in orphan genes with mitochondrial targeting peptides may signify an increased mitochondrial gene fostering activity. The correlation between orphan genes and predicted mitochondrial targeting is elucidated further by this analysis of orphan genes of 33 land plant species.

### Orphan Gene Content Is Correlated With Numt Content in Land Plant Genomes

**FIGURE 3:**
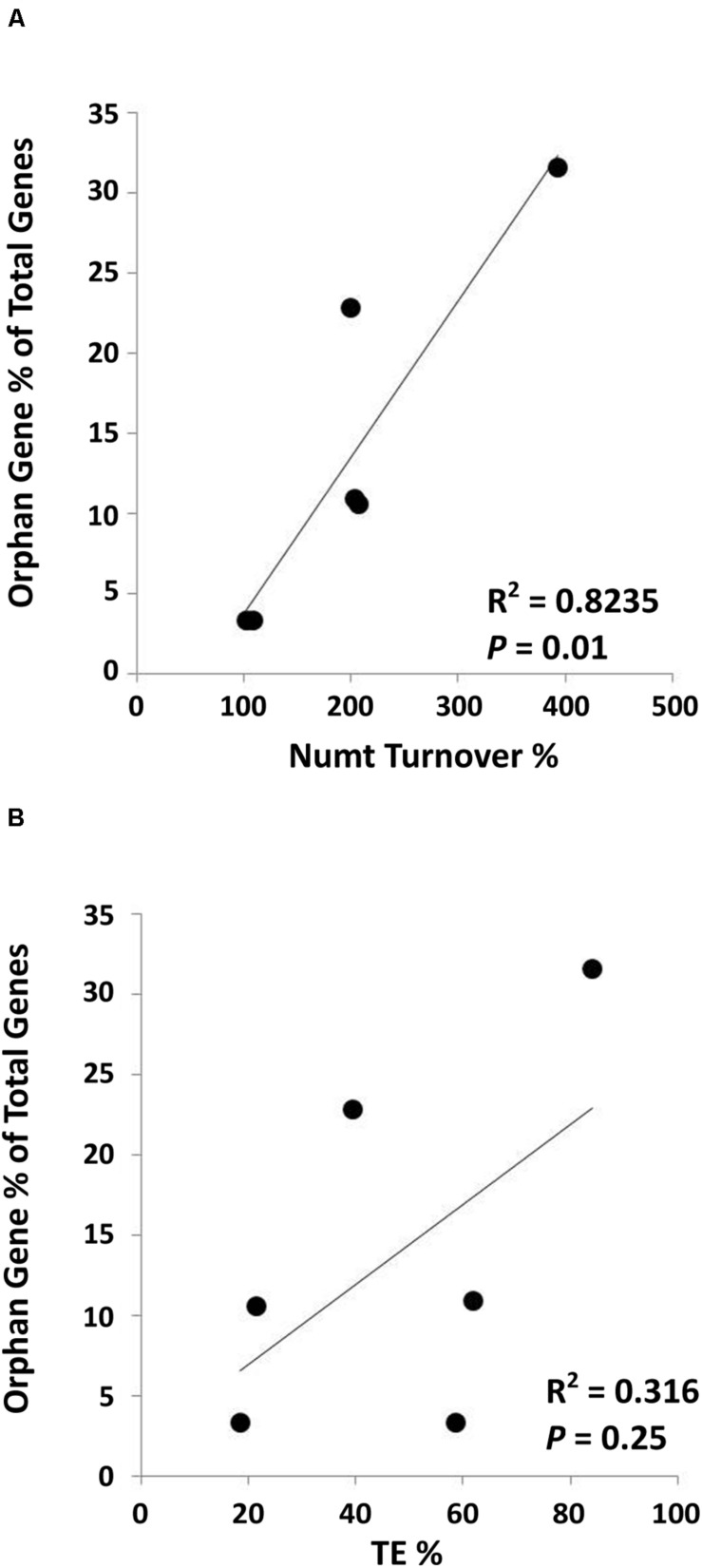
Positive correlation between Numts and orphan genes. **(A)** A significantly positive correlation between Numt turnover % (total Numt length/mitochondrial genome size) and orphan gene percentage (Correlation coefficient = 0.91; *P* = 0.01). **(B)** No significant correlation is found between % of TEs in the genome and orphan gene percentage (Correlation coefficient = 0.56; *P* = 0.25). Species used in this analysis*: A. thaliana*, *G. max*, *Z. mays*, *O. sativa*, *S. bicolor*, and *V. vinifera*. TE data was retrieved from Oliver et al. (2013). Orphan gene data for *O. sativa*, *Z. mays* and *S. bicolor* was obtained from Yao et al. (2017); *A. thaliana* was obtained from Arendsee et al. (2014); *G. max*, and *V. vinifera* were obtained from http://www.greenphyl.org/cgi-bin/index.cgi (version 4).

A collection of Numts for six different land plant species [Numt length data from Ko and Kim (2016)] was used to analyze the correlation between mitochondrial DNA transferred to the nucleus and the number of orphan genes present in these species. To do this, the whole Numt length (the total kilobases of every mitochondrial piece of DNA found in the nucleus) was divided by the length of the mitochondrial genome in that species then multiplied by 100 to give a value we termed the Numt turnover %. This tells us how much mitochondrial DNA was transferred in relation to the size of the mitochondrial genome in each species. We then used the total number of orphan genes and the total number of genes in each species to calculate the orphan gene content as a percent of total genes. These two numbers calculated for each of the six species were plotted and analyzed for correlation. Pearson correlation method returned a correlation coefficient of 0.91 with a *P* = 0.01 (*R*^2^ = 0.8235) (Figure 3A). This provides evidence that there is a positive correlation between the level of mitochondrial DNA transfer (Numt turnover %) and the proportion of orphan genes to total genes in a species (orphan gene %).

We also computed the correlation between orphan gene content in a genome and the percentage of TEs that make up the genome. A positive correlation was found, however, not statistically significant (correlation coefficient = 0.56, *P* = 0.25, and *R*^2^ = 0.316) (Figure 3B).

### Orphan Gene Content Is Not Correlated With Numt Content in Cetacean Species

**FIGURE 4:**
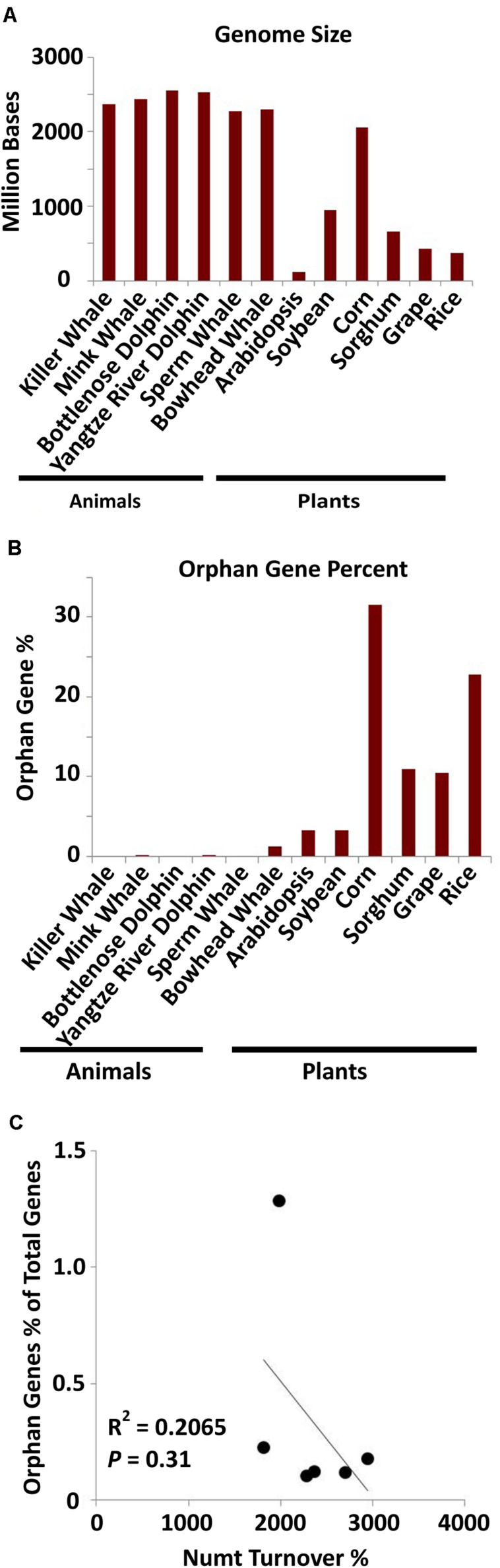
Orphan gene content in six aquatic mammals. **(A)** Whole genome sizes of the six mammalian species and the six plant species (data from NCBI). **(B)** Orphan gene content (as a percentage of total genes) for aquatic mammals and land plants. **(C)** The correlation of orphan gene content as percentage of total genes and Numt turnover %. Correlation coefficient = –0.48; *P* = 0.31. The amount of orphan genes in mammal species is not significantly correlated with their Numt content.

As mitochondrial sizes and dynamics are vastly different between mammals and plants (Burger et al., 2003; Barr et al., 2005; Ko and Kim, 2016; Supplementary Figure 3), orphan gene analysis was performed for six cetacean species via a BLAST filtering technique (Donoghue et al., 2011). Although the mammals have larger whole genomes (Figure 4A), they have much smaller mitochondrial genomes than land plants (Ko and Kim, 2016), and a much smaller orphan gene content than land plants (Figure 4B). No correlation was detected between orphan gene content with Numt turnover in mammal genomes (Figure 4C). This further provides evidence that the mitochondrial genome may play a role in orphan gene development in land plants, while that role has likely been minimized or lost in animals due to their small mitochondrial genome size.

### Several *A. thaliana* Orphan Genes Exist in Both Mitochondrial Genome and Nuclear Genome

Of the 30 predicted mitochondrial orphan genes in *A. thaliana*, 28 of them have at least some homology to a nuclear chromosome (Table 2). Of these 28, 22 orphans have a 100% query cover to a nuclear chromosome which means the entire sequence can be detected in both mitochondrial genome and nuclear genome, thus it is possible that the sequence could have been transferred from the mitochondrial genome to the nuclear genome. Most of these transfers are seen in chromosome two—which may be a result of the recent, large transfer event where nearly the entire mitochondrial genome was transferred to the pericentromeric region of the chromosome (Stupar et al., 2001). Furthermore, 29 of the 30 have hits to mitochondrial genome in another species. This implies that the sequences may have originated in the mitochondrial genome, and were recently transferred to the nuclear genome of *A. thaliana*. Only one orphan had sequence homology to a nuclear genome in a species other than *A. thaliana*. This reinforced the idea that these sequences may have originated in the mitochondrial genome.

**TABLE 2:** Genome homology of orphans of mitochondrial genome in *A. thaliana*.

Gene ID	Nuclear chromosome location	Query cover (%)	Knitted	Segments in mitochondrion of other species
AtMg00130	2,3	9,20		Yes
AtMg00140	4	7		Yes
AtMg00200	1,2	19,100		Yes
AtMg00260	2,3	100,100	Yes	Yes
AtMg00320	2	100		Yes
AtMg00430	2	100	Yes	Yes
AtMg00440	2	100	Yes	Yes
AtMg00450	2	100		Yes
AtMg00470	N/A	N/A	Yes	Yes
AtMg00600	2	100	Yes	Yes
AtMg00630	N/A	N/A		Yes
AtMg00680	2	20		
AtMg00720	2	25		Yes
AtMg00740*	2,3,5	100,14,22		Yes
AtMg00840	2	100		Yes
AtMg00870	5	100		Yes
AtMg00880	2,5	100,100		Yes
AtMg00890	1,2	9,100		Yes
AtMg01000	2,3	100,20		Yes
AtMg01050	2	100		Yes
AtMg01100	2,5	100,18		Yes
AtMg01140	2	100	Yes	Yes
AtMg01150	2	100	Yes	Yes
AtMg01180	2	100		Yes
AtMg01240	2	100		Yes
AtMg01260	1,2,3,5	5,100,5,18		Yes
AtMg01290	2	100		Yes
AtMg01300	2	100		Yes
AtMg01370	2	24		Yes
AtMg01400	4	40		Yes

Nearly all predicted orphan genes in the mitochondria have either complete or partial sequence transfer to the nuclear genome of Arabidopsis. *AtMg00740 is the only gene that has sequence homology to a nuclear chromosome of a different species (A. alpina). All genes except AtMg00680 have some sort of sequence homology to another species mitochondrial genome, suggesting the sequence may have originated in the mitochondrial genome and has been recently transferred to the nuclear genome of A. thaliana. Orphans were marked as knitted if two different segments of the query were found in the same mitochondrial genome of a particular species (other than A. thaliana).

### Mitochondrial Fostering of At2g07667 (*KNIT*), a Young *A. thaliana* Gene

**FIGURE 5:**
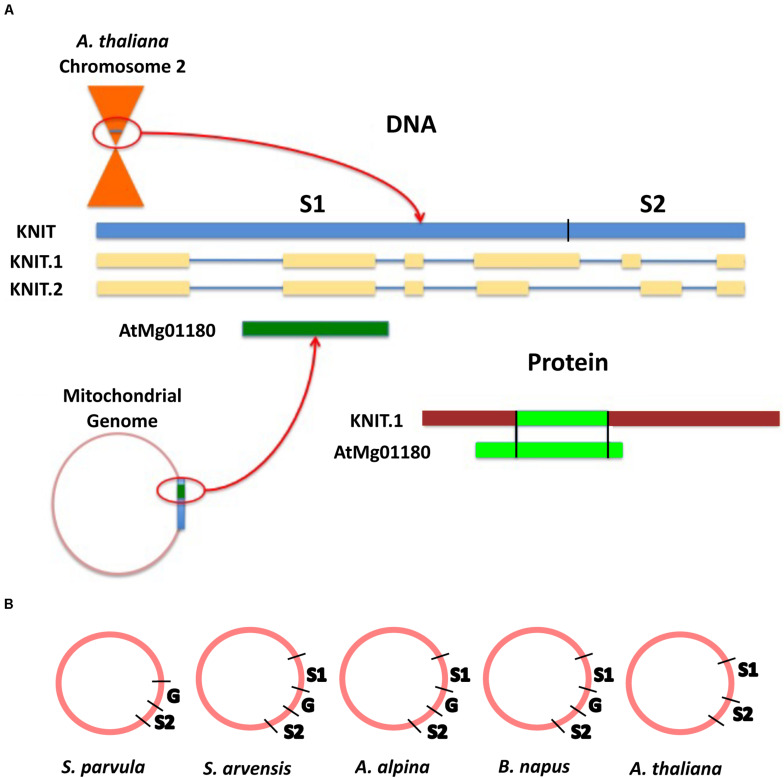
KNIT sequence origin and location. **(A)** Model of KNIT loci in both chromosome 2 and the mitochondrial genome. The gene model indicates that the full genomic sequence of KNIT exists in both chromosome 2 near the centromere and in the mitochondrial genome of *A. thaliana*, but the predicted protein sequences differ. **(B)** The gap sequence that separates genic sequences is not present in *A. thaliana*. G, gap sequence; S1, first segment of *KNIT* gene; S2, second segment of *KNIT* gene.

To better understand how orphan sequences may evolve in the mitochondrial genome, we analyzed a predicted nuclear orphan gene that has sequence homology to the mitochondrial genome of *A. thaliana*. Gene At2g07667, termed here as Knitted In Mitochondria (*KNIT*), is a protein-coding gene with two proposed gene models. There are six exons, and five introns in each of the models (Figure 5A). The protein consists of 218 amino acids and its full-length genomic sequence covers 1,746 base pairs (arabidopsis.org). Alignment analysis carried out with the NCBI BLASTn server indicated the nucleotide sequence of this gene is also found in the mitochondrial genome of *A. thaliana* (identity and query cover of 100%; Figure 5A). A BLASTp search revealed that the KNIT protein only had one potential homolog in *Brassica napus* (sequence ID: CDY45527.1), a protein made up of 171 amino acids with a query cover of 76% and an identity of 77%. tBLASTn analysis on the peptide sequence of the *B. napus* homolog shows that the protein maps back to the mitochondrial genome of *B. napus* covering 3 different ranges, suggesting at least two introns. This hit (BLASTp) to *B. napus* indicates that *KNIT* may not be a “pure” species-specific orphan gene, but is indeed a very young gene.

The only other protein sequence hit for the query KNIT belongs to the *A. thaliana* mitochondrial genome. However, even though the entire nucleotide sequence of *KNIT* is found in the mitochondrial genome, the gene annotated in this region is not identical to *KNIT*. In the mitochondrial genome, the gene locus ID AtMg01180 (Table 2), codes for a protein of 111 amino acids covering a portion of the first intron of KNIT, the entire second exon, and a portion of the second intron—covering amino acids 64–120 of KNIT protein (Figure 5A). Thus, different regions of the same nucleotide sequence are expressed between the mitochondrial and the nuclear genomes.

Alignment analysis (BLASTn) of KNIT showed hits throughout different mitochondrial genomes: *Schrenkiella parvula, Arabis alpina, Brassica napus, Sinapis arvensis*, and of course *A. thaliana* (Table 3). This suggests that the gene sequence originated in the mitochondrial genome. This sequence was possibly transferred to the *A. thaliana* chromosome two via a recent transfer event (Stupar et al., 2001). The first 1,290 nucleotides (Segment 1) of KNIT can be found in all above species except *S. parvula*, and the second 475 nucleotides (Segment 2) is found in all 5 species. However, these two gene segments are separated by a 652-nucleotide segment (Gap) in *A. alpine, B. napus*, and *S. arvensis*. This Gap sequence is not present in *A. thaliana*, but is present in *S. parvula* as well (Figure 5B). Along with KNIT, seven of the mitochondrial orphans show clear knitting patterns—multiple segments of the query were found in multiple locations in the mitochondrial genome of another species (Table 2).

**TABLE 3:** Alignment analysis for KNIT (BLASTn hit).

Query name	Subject name	Segment	Identity	Alignment length	Unaligned bases
*KNIT*	*A. thaliana* ecotype Col-0 mitochondrion	KNIT Full	100	1,746	0
*KNIT*	*S. arvensis* mitochondrion	Segment 1	99.61	1,286	5
*KNIT*	*S. arvensis* mitochondrion	Segment 2	99.58	475	2
*KNIT*	*B. napus* strain 56366 mitochondrion	Segment 1	98.60	1,289	18
*KNIT*	*B. napus* strain 56366 mitochondrion	Segment 2	99.16	475	4
*KNIT*	*A. alpina* mitochondrion	Segment 1	97.91	1,290	27
*KNIT*	*A. alpina* mitochondrion	Segment 2	98.95	475	5
*KNIT*	*S. parvula* mitochondrion	Segment 2	98.95	475	5

*One hit was reported in A. thaliana (outside of the chromosome 2 hit) with a 100% match spanning all 1,746 nucleotides. For S. arvensis, B. napus, and A. alpina, two individual hits (one around 1,290 nt and the other 475 nt) to the mitochondrial genome were reported. S. parvula had one hit (475 nt) reported. A small amount of unaligned bases for all hits underlie a low mutation rate of the mitochondrial genomes.*

**FIGURE 6:**
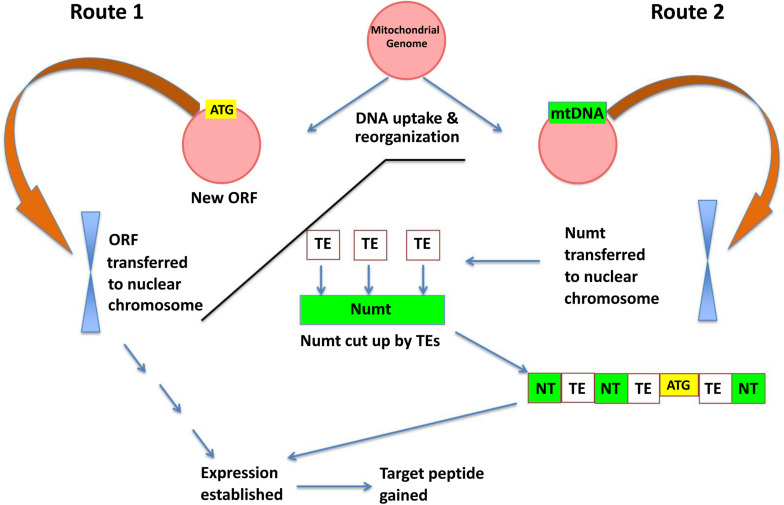
A model for orphan gene evolution through the mitochondrial genome. Novel sequences are created due to the high rearrangement rate in the mitochondrial genome, and then inserted into a nuclear chromosome. The transferred DNA may already contain gene coding information like *KNIT* (left side of model: Route 1) or may obtain an open reading frame via other genome mechanisms such as transposon transposition (right side: Route 2).

To explain the possible impact of the mitochondrial genome on orphan gene evolution, we propose a model with two routes whereby orphan open reading frames may be either: (1) generated in the mitochondrial genome and transferred to the nuclear genome as a Numt or (2) mitochondrial DNA is transferred to the nuclear genome and further diversification (e.g., cutting by TEs, integration with nuclear DNA, mutations, etc.) generates novel open reading frames (Figure 6). Based on the large percentage of orphan genes in plant mitochondria, and evidence that these open reading frames can transfer among genomes, it is likely that Route 1 may play a role in the evolution of novel genic content in the nuclear genome.

## Discussion

Our analysis of orphan gene content in three well annotated plant mitochondrial genomes found the mitochondrial genome contained a higher proportion of orphan genes compared to the chloroplast genome and whole genome (Figure 1). This is likely because of the high recombination rates and sequence diversity of the plant mitochondrial genome (Christensen, 2013). As large mitochondrial sequences are often transferred to the nucleus (Lopez et al., 1996; Bensasson et al., 2001; Stupar et al., 2001; Richly and Leister, 2004; Noutsos et al., 2005; Michalovova et al., 2013; Ko et al., 2015; Zhang et al., 2020), looking into the relationship between Numts and orphan genes may prove fruitful in understanding *de novo* gene evolution.

As the original transfer of mitochondrial sequences to the nuclear genome led to a mass organelle gene exodus that required the transferred genes to obtain targeting back to the organelle (Berg and Kurland, 2000), we examined whether current orphan genes in *A. thaliana* preferentially target mitochondria. Indeed, we found that a large proportion of orphan genes are predicted to target mitochondria (Figure 2). We next determined if mitochondrial originating DNA (Numts) preferentially code for mitochondrial targeting peptides compared to intergenic DNA. We found that mitochondrial originating DNA does preferentially code for a mitochondrial targeting peptide (Figure 2). This further leads us to believe that it is possible for orphan genes in *A. thaliana* to arise from mitochondrial originating DNA. Mitochondrial originating DNA and completely randomized DNA sequences each preferentially code for mitochondrial targeting peptides, suggesting that Numts have a random quality, possibly allowing for the creation of novel genes with no known functional motifs, a trait common to orphan genes (Arendsee et al., 2014; Figure 2).

When looking at the genomes as a whole, mammal genomes are much larger, while their mitochondrial genomes are much smaller than plant mitochondrial genomes (Figure 4A). We can also see that plant mitochondrial genomes are much more dynamic in size between the organisms whereas mammal mitochondrial genomes are nearly identical in size (Supplementary Figure 3). Plants also have more Numts on average than animals (Bensasson et al., 2001), and they appear to be more dynamic in size as well (Ko and Kim, 2016). Therefore, mammal mitochondrial genomes, having lost size and dynamic nature, likely do not play as much of an active role in orphan gene evolution, hence the smaller amount of orphan genes and lack of correlation with Numts compared to plant mitochondrial genomes (Figures 3, 4).

To determine if full orphan gene sequences can transfer from the mitochondrial genome to the nuclear genome, sequence alignment analysis was performed for all 30 predicted mitochondrial orphan genes in *A. thaliana*. The vast majority of these orphan sequences were also found in the nuclear chromosomes of *A. thaliana*. Hits to species other than *A. thaliana* were limited to the mitochondrial genomes (Table 2). This indicates these orphans may have been formed in the mitochondrial genome and their sequences were recently transferred to the nuclear genome.

In order to better understand how the mitochondrial genome can evolve orphan genes and subsequently transfer them to the nuclear genome, an orphan gene with a sequence in both the mitochondrial genome and chromosome two was chosen for sequence analysis. This analysis appears to show the evolution of *KNIT* as a result of reshuffling in the mitochondrial genome of *A. thaliana* (Figure 5) and a subsequent transfer to chromosome 2. The low number of SNPs (single nucleotide polymorphism) between the partial *KNIT* sequences in the mitochondria of *A. thaliana* and other species (Table 3) further demonstrates that the creative power of the mitochondrial genome is in its rearrangement ability and not its mutation rate. This also demonstrates that mitochondrial orphan genes are not protected from genome rearrangement like conserved genes are, so the evolutionary potential in a mitochondrial orphan gene may be much higher once transferred to the nuclear genome—where there are fewer rearranging events. This process seems to be a sort of evolutionary communication between the mitochondrial genome and the nuclear genome. In addition, like *KNIT*, several other mitochondrial orphan genes have evidence of being knitted in the mitochondrial genome due to genome rearrangements (Table 2).

## Conclusion

Based on this work and the current knowledge behind plant mitochondrial genome dynamics, we propose a model whereby novel sequences, sometimes containing orphan genes, may be generated in the plant mitochondrial genomes based on uptake of DNA from the nucleus/chloroplast/horizontal gene transfer and subsequent reshuffling of the organelle genome. When large Numts are transferred to the nuclear genome, the novel sequences are transferred as well and are then subject to evolutionary mechanisms in the nuclear genome (higher mutation rate/enhanced epigenetic control/TEs/etc.). This process of intracellular genome crosstalk, which we call mitochondrial fostering, may play an integral role in the evolution of *de novo* orphan genes in plants.

## Data Availability Statement

The datasets presented in this study can be found in online repositories. The names of the repository/repositories and accession number(s) can be found in the article/Supplementary Material.

## Author Contributions

SO’C conceived the study, designed and performed the experiments, analyzed the data, and wrote the manuscript. LL conceived the study, designed the experiments, and wrote the manuscript. Both authors contributed to the article and approved the submitted version.

## Conflict of Interest

The authors declare that the research was conducted in the absence of any commercial or financial relationships that could be construed as a potential conflict of interest.

## Footnotes

1 http://bioinformatics.org/sms2/split_fasta.html

2 http://www.cbs.dtu.dk/services/TargetP-1.1/index.php

3 https://www.soybase.org/soytedb/

4 https://www.uniprot.org/uniprot/

5 http://www.bowhead-whale.org/

6 http://www.greenphyl.org/cgi-bin/index.cgi

## References

[B1] Arendsee Z. W. Li L. Wurtele E. S. (2014). Coming of age: orphan genes in plants. *Trends Plant Sci.* 19 698–708. 10.1016/j.tplants.2014.07.003 25151064

[B2] Arrieta-Montiel M. P. Shedge V. Davila J. Christensen A. C. Mackenzie S. A. (2009). Diversity of the *Arabidopsis* mitochondrial genome occurs via nuclear-controlled recombination activity. *Genetics* 183 1261–1268. 10.1534/genetics.109.108514 19822729 PMC2787419

[B3] Barr C. M. Neiman M. Taylor D. R. (2005). Inheritance and recombination of mitochondrial genomes in plants, fungi and animals. *New Phytol.* 168 39–50. 10.1111/j.1469-8137.2005.01492.x 16159319

[B4] Bennetzen J. L. (2005). Transposable elements, gene creation and genome rearrangement in flowering plants. *Curr. Opin. Genet. Dev.* 15 621–627. 10.1016/j.gde.2005.09.010 16219458

[B5] Bensasson D. Zhang D.-X. Hartl D. L. Hewitt G. M. (2001). Mitochondrial pseudogenes: evolution’s misplaced witnesses. *Trends Ecol. Evol.* 16 314–321. 10.1016/S0169-5347(01)02151-6 11369110

[B6] Berg O. G. Kurland C. G. (2000). Why mitochondrial genes are most often found in nuclei. *Mol. Biol. Evol.* 17 951–961. 10.1093/oxfordjournals.molbev.a026376 10833202

[B7] Buchfink B. Xie C. Huson D. H. (2015). Fast and sensitive protein alignment using DIAMOND. *Nat. Methods* 12 59–60. 10.1038/nmeth.3176 25402007

[B8] Burger G. Gray M. W. Franz Lang B. (2003). Mitochondrial genomes: anything goes. *Trends Genet.* 19 709–716. 10.1016/j.tig.2003.10.012 14642752

[B9] Calabrese F. M. Balacco D. L. Preste R. Diroma M. A. Forino R. Ventura M. (2017). NumtS colonization in mammalian genomes. *Sci. Rep.* 7:16357. 10.1038/s41598-017-16750-2 29180746 PMC5703718

[B10] Christensen A. C. (2013). Plant mitochondrial genome evolution can be explained by DNA repair mechanisms. *Genome Biol. Evol.* 5 1079–1086. 10.1093/gbe/evt069 23645599 PMC3698917

[B11] Donoghue M. T. A. Keshavaiah C. Swamidatta S. H. Spillane C. (2011). Evolutionary origins of Brassicaceae specific genes in *Arabidopsis thaliana*. *BMC Evol. Biol.* 11:47. 10.1186/1471-2148-11-47 21332978 PMC3049755

[B12] Dujon B. (1996). The yeast genome project: what did we learn? *Trends Genet.* 12 263–270. 10.1016/0168-9525(96)10027-5 8763498

[B13] Ekstrom A. Yin Y. (2016). ORFanFinder: automated identification of taxonomically restricted orphan genes. *Bioinformatics* 32 2053–2055. 10.1093/bioinformatics/btw122 27153690 PMC4920126

[B14] Emanuelsson O. Nielsen H. Brunak S. Von Heijne G. (2000). Predicting subcellular localization of proteins based on their N-terminal amino acid sequence. *J. Mol. Biol.* 300 1005–1016. 10.1006/jmbi.2000.3903 10891285

[B15] Graham M. A. Silverstein K. A. T. Cannon S. B. Vandenbosch K. A. (2004). Computational identification and characterization of novel genes from legumes. *Plant Physiol.* 135 1179–1197. 10.1104/pp.104.037531 15266052 PMC519039

[B16] Hazkani-Covo E. Sorek R. Graur D. (2003). Evolutionary dynamics of large numts in the human genome: rarity of independent insertions and abundance of post-insertion duplications. *J. Mol. Evol.* 56 169–174. 10.1007/s00239-002-2390-5 12574863

[B17] Hazkani-Covo E. Zeller R. M. Martin W. (2010). Molecular poltergeists: mitochondrial DNA copies (numts) in sequenced nuclear genomes. *PLoS Genet.* 6:e1000834. 10.1371/journal.pgen.1000834 20168995 PMC2820518

[B18] Jensen-Seaman M. I. Wildschutte J. H. Soto-Calderón I. D. Anthony N. M. (2009). A comparative approach shows differences in patterns of numt insertion during hominoid evolution. *J. Mol. Evol.* 68 688–699. 10.1007/s00239-009-9243-4 19471988 PMC3140062

[B19] Jones D. C. Zheng W. Huang S. Du C. Zhao X. Yennamalli R. M. (2016). A clade-specific *Arabidopsis* gene connects primary metabolism and senescence. *Front. Plant Sci.* 7:983. 10.3389/fpls.2016.00983 27462324 PMC4940393

[B20] Ko Y.-J. Kim S. (2016). Analysis of nuclear mitochondrial DNA segments of nine plant species: size, distribution, and insertion loci. *Genomics Inform.* 14 90–95. 10.5808/GI.2016.14.3.90 27729838 PMC5056902

[B21] Ko Y.-J. Yang E. C. Lee J.-H. Lee K. W. Jeong J.-Y. Park K. (2015). Characterization of cetacean Numt and its application into cetacean phylogeny. *Genes Genomics* 37 1061–1071. 10.1007/s13258-015-0353-7

[B22] Kubo T. Mikami T. (2007). Organization and variation of angiosperm mitochondrial genome. *Physiol. Plant.* 129 6–13. 10.1111/j.1399-3054.2006.00768.x

[B23] Kumar A. Gates P. B. Czarkwiani A. Brockes J. P. (2015). An orphan gene is necessary for preaxial digit formation during salamander limb development. *Nat. Commun.* 6:8684. 10.1038/ncomms9684 26498026 PMC4918474

[B24] Li L. Foster C. M. Gan Q. Nettleton D. James M. G. Myers A. M. (2009). Identification of the novel protein QQS as a component of the starch metabolic network in *Arabidopsis* leaves. *Plant J.* 58 485–498. 10.1111/j.1365-313X.2009.03793.x 19154206

[B25] Li L. Wurtele E. S. (2015). The QQS orphan gene of *Arabidopsis* modulates carbon and nitrogen allocation in soybean. *Plant Biotechnol. J.* 13 177–187. 10.1111/pbi.12238 25146936 PMC4345402

[B26] Li L. Zheng W. Zhu Y. Ye H. Tang B. Arendsee Z. W. (2015). QQS orphan gene regulates carbon and nitrogen partitioning across species via NF-YC interactions. *Proc. Natl. Acad. Sci. U.S.A.* 112 14734–14739. 10.1073/pnas.1514670112 26554020 PMC4664325

[B27] Lopez J. V. Cevario S. O’brien S. J. (1996). Complete nucleotide sequences of the domestic cat (Felis catus) mitochondrial genome and a transposed mtDNA tandem repeat (Numt) in the nuclear genome. *Genomics* 33 229–246. 10.1006/geno.1996.0188 8660972

[B28] Luhua S. Hegie A. Suzuki N. Shulaev E. Luo X. Cenariu D. (2013). Linking genes of unknown function with abiotic stress responses by high-throughput phenotype screening. *Physiol. Plant* 148 322–333. 10.1111/ppl.12013 23517122

[B29] Michalovova M. Vyskot B. Kejnovsky E. (2013). Analysis of plastid and mitochondrial DNA insertions in the nucleus (NUPTs and NUMTs) of six plant species: size, relative age and chromosomal localization. *Heredity (Edinb)* 111 314–320. 10.1038/hdy.2013.51 23715017 PMC3807264

[B30] Noutsos C. Kleine T. Armbruster U. Dalcorso G. Leister D. (2007). Nuclear insertions of organellar DNA can create novel patches of functional exon sequences. *Trends Genet.* 23 597–601. 10.1016/j.tig.2007.08.016 17981356

[B31] Noutsos C. Richly E. Leister D. (2005). Generation and evolutionary fate of insertions of organelle DNA in the nuclear genomes of flowering plants. *Genome Res.* 15 616–628. 10.1101/gr.3788705 15867426 PMC1088290

[B32] O’Conner S. Li L. (2020). Mitochondrial fostering: the mitochondrial genome may play a role in plant orphan gene evolution. *Biorxiv [Preprint]* 10.1101/2020.01.02.884874 PMC7793901 33424897

[B33] O’Conner S. Neudorf A. Zheng W. Qi M. Zhao X. Du C. (2018). “From *Arabidopsis* to crops: the *Arabidopsis* QQS orphan gene modulates nitrogen allocation across species,” in *Engineering Nitrogen Utilization in Crop Plants Target traits*, eds Shrawat A. Zayed A. Lightfoot D. A. (Cham: Springer International Publishing), 95–117. 10.1007/978-3-319-92958-3_6

[B34] Oliver K. R. McComb J. A. Greene W. K. (2013). Transposable elements: powerful contributors to angiosperm evolution and diversity. *Genome Biol. Evol.* 5 1886–1901. 10.1093/gbe/evt141 24065734 PMC3814199

[B35] Qi M. Zheng W. Zhao X. Hohenstein J. D. Kandel Y. O’conner S. (2019). QQS orphan gene and its interactor NF-YC4 reduce susceptibility to pathogens and pests. *Plant Biotechnol. J.* 17 252–263. 10.1111/pbi.12961 29878511 PMC6330549

[B36] Quinlan A. R. Hall I. M. (2010). BEDTools: a flexible suite of utilities for comparing genomic features. *Bioinformatics (Oxford, England)* 26 841–842. 10.1093/bioinformatics/btq033 20110278 PMC2832824

[B37] Reinhardt J. A. Wanjiru B. M. Brant A. T. Saelao P. Begun D. J. Jones C. D. (2013). De Novo ORFs in drosophila are important to organismal fitness and evolved rapidly from previously non-coding sequences. *PLoS Genet.* 9:e1003860. 10.1371/journal.pgen.1003860 24146629 PMC3798262

[B38] Rice D. W. Alverson A. J. Richardson A. O. Young G. J. Sanchez-Puerta M. V. Munzinger J. (2013). Horizontal transfer of entire genomes via mitochondrial fusion in the angiosperm *Amborella*. *Science* 342 1468–1473. 10.1126/science.1246275 24357311

[B39] Richly E. Leister D. (2004). NUMTs in sequenced eukaryotic genomes. *Mol. Biol. Evol.* 21 1081–1084. 10.1093/molbev/msh110 15014143

[B40] Rödelsperger C. (2018). Comparative genomics of gene loss and gain in *Caenorhabditis* and other nematodes. *Methods Mol. Biol.* 1704 419–432. 10.1007/978-1-4939-7463-4_16 29277876

[B41] Sanchez-Puerta M. V. Cho Y. Mower J. P. Alverson A. J. Palmer J. D. (2008). Frequent, phylogenetically local horizontal transfer of the *cox1* group I Intron in flowering plant mitochondria. *Mol. Biol. Evol.* 25 1762–1777. 10.1093/molbev/msn129 18524785 PMC2727383

[B42] Skippington E. Barkman T. J. Rice D. W. Palmer J. D. (2015). Miniaturized mitogenome of the parasitic plant *Viscum scurruloideum* is extremely divergent and dynamic and has lost all nad genes. *Proc. Natl. Acad. Sci. U. S. A.* 112 E3515–E3524. 10.1073/pnas.1504491112 26100885 PMC4500244

[B43] Sloan D. B. Alverson A. J. Chuckalovcak J. P. Wu M. Mccauley D. E. Palmer J. D. (2012). Rapid evolution of enormous, multichromosomal genomes in flowering plant mitochondria with exceptionally high mutation rates. *PLoS Biol.* 10:e1001241. 10.1371/journal.pbio.1001241 22272183 PMC3260318

[B44] Stupar R. M. Lilly J. W. Town C. D. Cheng Z. Kaul S. Buell C. R. (2001). Complex mtDNA constitutes an approximate 620-kb insertion on *Arabidopsis thaliana* chromosome 2: implication of potential sequencing errors caused by large-unit repeats. *Proc. Natl. Acad. Sci. U. S. A.* 98 5099–5103. 10.1073/pnas.091110398 11309509 PMC33170

[B45] Tautz D. Domazet-Lošo T. (2011). The evolutionary origin of orphan genes. *Nat. Rev. Genet.* 12 692–702. 10.1038/nrg3053 21878963

[B46] Timmis J. N. Ayliffe M. A. Huang C. Y. Martin W. (2004). Endosymbiotic gene transfer: organelle genomes forge eukaryotic chromosomes. *Nat. Rev. Genet.* 5 123–135. 10.1038/nrg1271 14735123

[B47] Triant D. A. DeWoody J. A. (2007). Extensive mitochondrial DNA transfer in a rapidly evolving rodent has been mediated by independent insertion events and by duplications. *Gene* 401 61–70. 10.1016/j.gene.2007.07.003 17714890

[B48] Woischnik M. Moraes C. T. (2002). Pattern of organization of human mitochondrial pseudogenes in the nuclear genome. *Genome Res.* 12 885–893. 10.1101/gr.227202 12045142 PMC1383742

[B49] Wynn E. L. Christensen A. C. (2019). Repeats of unusual size in plant mitochondrial genomes: identification, incidence and evolution. *G3 (Bethesda)* 9 549–559. 10.1534/g3.118.200948 30563833 PMC6385970

[B50] Xi Z. Wang Y. Bradley R. K. Sugumaran M. Marx C. J. Rest J. S. (2013). Massive mitochondrial gene transfer in a parasitic flowering plant clade. *PLoS Genet.* 9:e1003265. 10.1371/journal.pgen.1003265 23459037 PMC3573108

[B51] Yao C. Yan H. Zhang X. Wang R. (2017). A database for orphan genes in Poaceae. *Exp. Ther. Med.* 14 2917–2924. 10.3892/etm.2017.4918 28966675 PMC5615222

[B52] Zhang G.-J. Dong R. Lan L.-N. Li S.-F. Gao W.-J. Niu H.-X. (2020). Nuclear Integrants of Organellar DNA contribute to genome structure and evolution in plants. *Int. J. Mol. Sci.* 21:707. 10.3390/ijms21030707 31973163 PMC7037861

[B53] Zhao N. Wang Y. Hua J. (2018). The roles of mitochondrion in intergenomic gene transfer in plants: a source and a pool. *Int. J. Mol. Sci.* 19:547. 10.3390/ijms19020547 29439501 PMC5855769

