# Supplementary material for: Mitochondrial Fostering: The Mitochondrial Genome May Play a Role in Plant Orphan Gene Evolution
Source: Front Plant Sci. 2020 Dec 3;11:600117. doi: 10.3389/fpls.2020.600117 (PMC7793901; doi:10.3389/fpls.2020.600117)
Supplement: Supplementary file 1 [file Data_Sheet_1.docx]

**Supplemental Material for**

**Mitochondrial fostering: the mitochondrial genome may play a role in plant orphan gene evolution**

**Seth O’Conner^1^ and Ling Li^1*^**

^1^Department of Biological Sciences, Mississippi State University, Mississippi State, MS, USA

**^*^Correspondence:**

Ling Li

[liling@biology.msstate.edu](mailto:liling@biology.msstate.edu)

**Supplementary Figures**

**
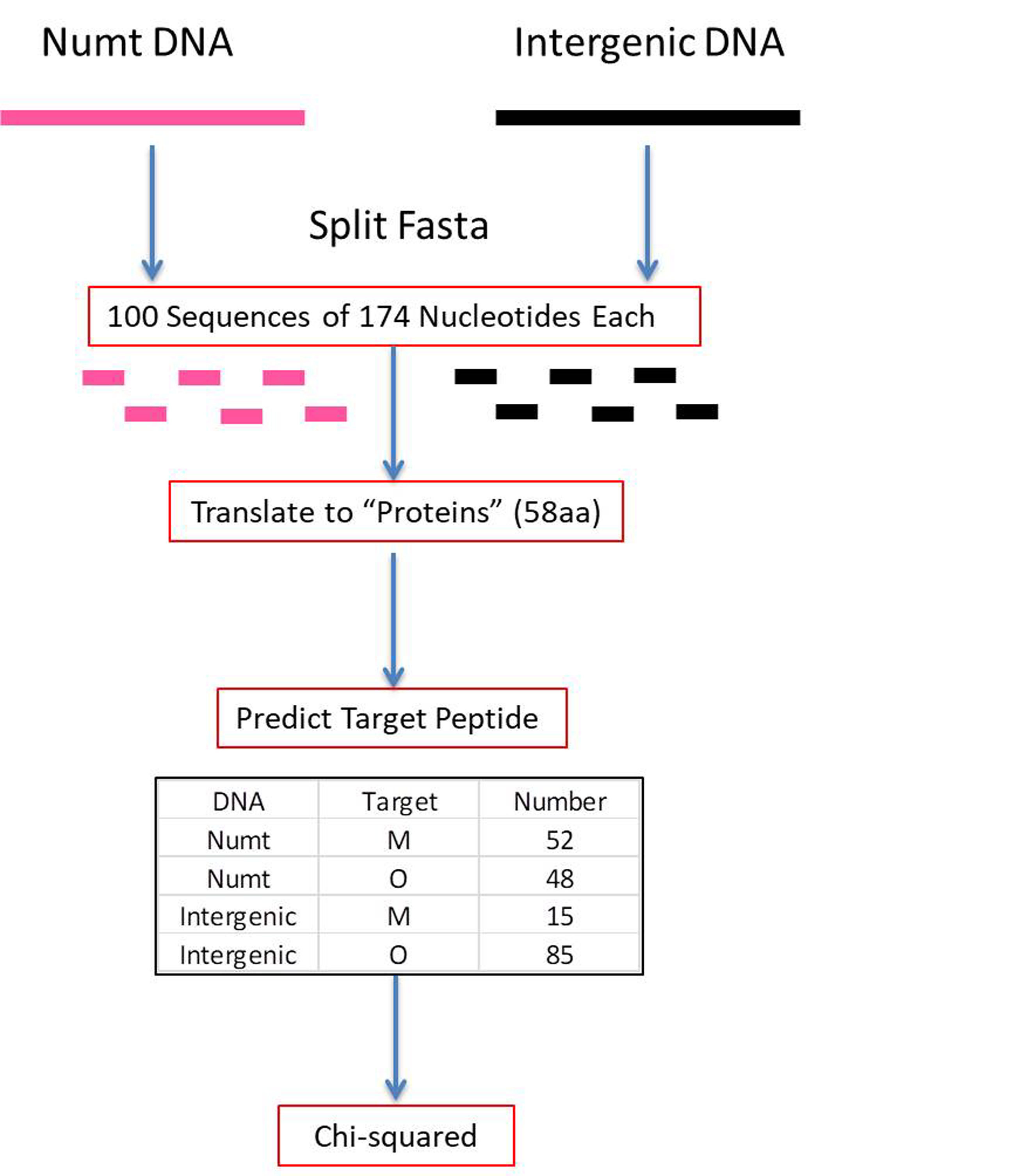
**

**Figure 1. Procedural flow chart for Targeting Peptide analysis.** A large section of Numt DNA sequence and intergenic DNA sequence was selected and split into 100 smaller sequences of 174 nucleotides each. These sequences were translated as if they were protein coding in order to get our “proteins”. Targeting peptide analysis was performed followed by statistical analysis. This procedure was aimed at answering this question: if orphan reading frames were to evolve from these sequence contexts, what would their predicted target peptide be?

**
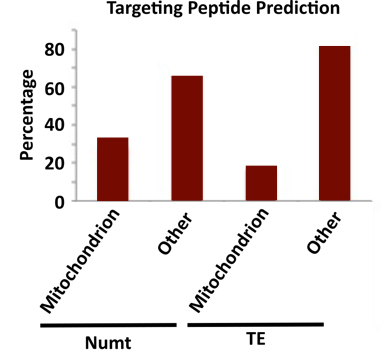
**

**Figure 2. Numt DNA in *G. max* is more likely to code for a mitochondrial targeting peptide as compared to transposable element DNA.** This is as similar as in Arabidopsis, Chi-squared *P* = 0.00593. One hundred sixty-four sequences of 174 nt were randomly sampled for *G. max* Numt and transposable element DNAs, and subsequently run through TargetP.


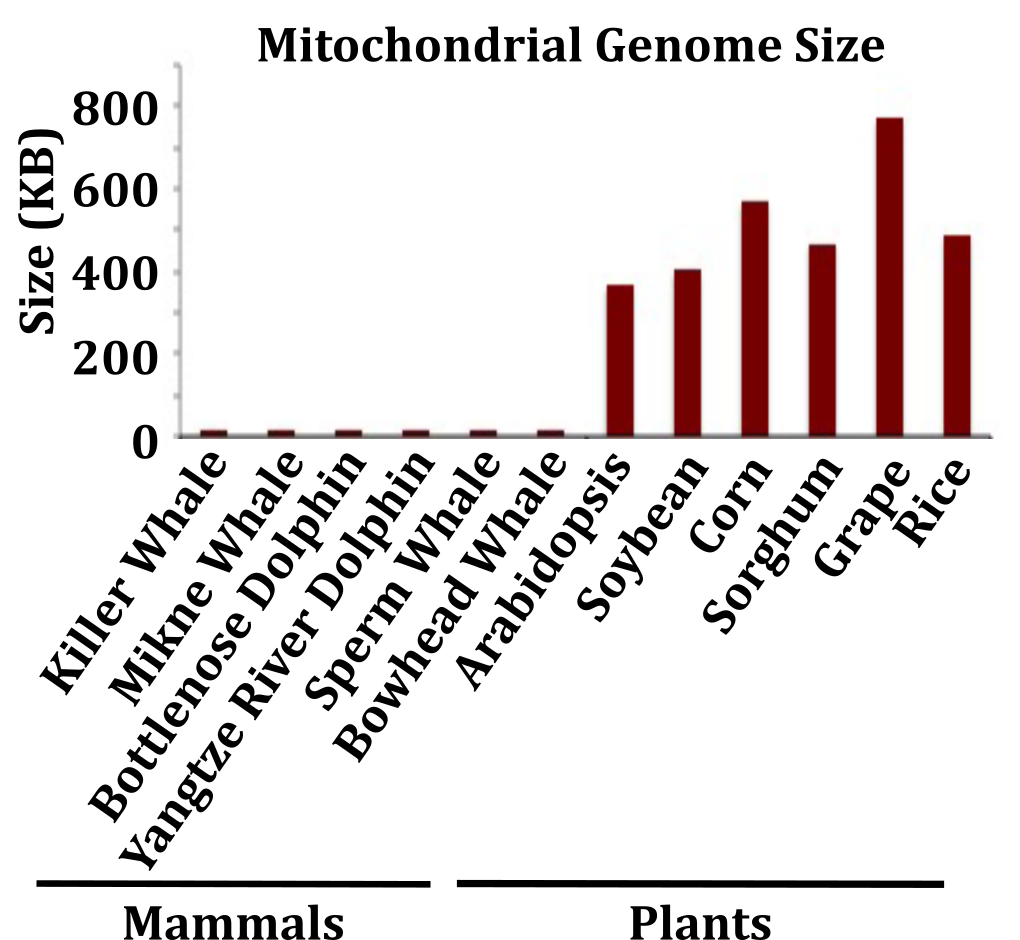


**Figure 3.** **Mitochondrial genomes are much larger in plant species compared to the mammal species used in this study.** Mitochondrial genome size for six mammal species and six plant species.

**Table 1| 33 plant orphan gene content and putative targeting peptides.**

| **Organism** | **Chloroplast (%)** | **Mitochondria (%)** | **Secreted (%)** | **Orphans (%)** |
| --- | --- | --- | --- | --- |
| *A. thaliana* | 5.06 | 17.11 | 22.10 | 4.36 |
| *G. max* | 10.62 | 18.59 | 32.28 | 3.29 |
| *O. sativa* | 18.74 | 26.50 | 10.55 | 10.00 |
| *V. vinifera* | 9.65 | 20.51 | 23.24 | 10.53 |
| *S. bicolor* | 36.16 | 26.55 | 10.73 | 0.60 |
| *Z. mays* | 23.26 | 29.06 | 14.47 | 8.25 |
| *Cicer* | 17.50 | 13.90 | 11.78 | 0.99 |
| *A. trichopoda* | 15.10 | 21.40 | 8.40 | 14.78 |
| *B. distachyon* | 13.10 | 21.30 | 17.00 | 1.72 |
| *C. cajans* | 23.40 | 18.70 | 13.50 | 0.56 |
| *C. papaya* | 11.51 | 22.64 | 8.11 | 12.42 |
| *C. sinensis* | 11.90 | 20.20 | 24.50 | 2.13 |
| *C. arabica* | 11.50 | 20.90 | 26.50 | 4.75 |
| *C. sativus* | 13.19 | 21.00 | 25.00 | 4.17 |
| *E. guineensis* | 17.90 | 17.90 | 10.30 | 0.11 |
| *G. raimondii* | 14.60 | 18.40 | 28.10 | 1.67 |
| *H. vulgare* | 5.60 | 22.20 | 33.30 | 0.07 |
| *L. japonicus* | 11.20 | 23.60 | 19.40 | 10.30 |
| *M. domestica* | 12.80 | 21.60 | 19.50 | 7.02 |
| *M. esculenta* | 14.80 | 21.80 | 21.60 | 1.39 |
| *M. truncatula* | 8.90 | 19.80 | 15.50 | 9.93 |
| *M. acuminata* | 11.80 | 22.90 | 21.00 | 12.22 |
| *M. balbisiana* | 10.20 | 17.80 | 11.90 | 4.39 |
| *P. vulgaris* | 13.90 | 16.20 | 22.10 | 1.43 |
| *P. dactylifera* | 6.90 | 16.20 | 10.40 | 4.00 |
| *P. patens* | 13.70 | 27.20 | 12.50 | 25.97 |
| *P. abies* | 9.00 | 16.80 | 9.70 | 12.45 |
| *P. tremula* | 10.60 | 20.00 | 25.10 | 4.06 |
| *R. communis* | 11.23 | 24.80 | 11.30 | 18.98 |
| *S. moellendorffii* | 11.10 | 17.00 | 15.80 | 4.71 |
| *S. italica* | 18.20 | 22.20 | 12.10 | 5.05 |
| *S. lycocarpum* | 8.70 | 20.30 | 27.60 | 8.46 |
| *T. cacao* | 6.20 | 18.40 | 24.50 | 3.01 |

*All orphan gene sequences for all 33 plants were collected from greenphyl version 4.*
